# Supplementary material for: A Critical View of the Application of the APEX Software (Aqueous Photochemistry of Environmentally-Occurring Xenobiotics) to Predict Photoreaction Kinetics in Surface Freshwaters
Source: Molecules. 2019 Dec 18;25(1):9. doi: 10.3390/molecules25010009 (PMC7017383; doi:10.3390/molecules25010009)
Supplement: Supplementary file 1 [file molecules-25-00009-s001.zip › 3.2.4_gcc-4.4.0.zip!/3.2.4_gcc-4.4.0/3.2.4_gcc-4.4.0/doc/octave/html/interpreter/Numeric-Input-Conversions.html]

Numeric Input Conversions - Untitled


Next: String Input Conversions,
Previous: Table of Input Conversions,
Up: C-Style I/O Functions


---

#### 14.2.14 Numeric Input Conversions

This section describes the `scanf` conversions for reading numeric
values.

The %d conversion matches an optionally signed integer in decimal
radix.

The %i conversion matches an optionally signed integer in any of
the formats that the C language defines for specifying an integer
constant.

For example, any of the strings 10, 0xa, or 012
could be read in as integers under the %i conversion. Each of
these specifies a number with decimal value `10`.

The %o, %u, and %x conversions match unsigned
integers in octal, decimal, and hexadecimal radices, respectively.

The %X conversion is identical to the %x conversion. They
both permit either uppercase or lowercase letters to be used as digits.

Unlike the C language `scanf`, Octave ignores the h,
l, and L modifiers.
